# Process Evaluation of a Medical Student–Delivered Smoking Prevention Program for Secondary Schools: Protocol for the Education Against Tobacco Cluster Randomized Trial

**DOI:** 10.2196/13508

**Published:** 2019-04-11

**Authors:** Titus Josef Brinker, Fabian Buslaff, Janina Leonie Suhre, Marc Philipp Silchmüller, Evgenia Divizieva, Jilada Wilhelm, Gabriel Hillebrand, Dominik Penka, Benedikt Gaim, Susanne Swoboda, Sonja Baumermann, Jörg Werner Walther, Christian Martin Brieske, Lena Jakob, Hannah Maria Baumert, Ole Anhuef, Selina Marisa Schmidt, Jonas Alfitian, Anil Batra, Lava Taha, Ute Mons, Felix Johannes Hofmann, Ailís Ceara Haney, Caelán Max Haney, Samuel Schaible, Thien-An Tran, Hanna Beißwenger, Tobias Stark, David A Groneberg, Werner Seeger, Aayushi Srivastava, Henning Gall, Julia Holzapfel, Nancy A Rigotti, Tanja Gabriele Baudson, Alexander H Enk, Stefan Fröhling, Christof von Kalle, Breno Bernardes-Souza, Rayanna Mara de Oliveira Santos Pereira, Roger Thomas

**Affiliations:** 1 Department of Translational Oncology National Center for Tumor Diseases, German Cancer Research Center (DKFZ) University of Heidelberg Heidelberg Germany; 2 Department of Dermatology University Hospital Heidelberg Heidelberg Germany; 3 Faculty of Medicine University of Bonn Bonn Germany; 4 Faculty of Medicine University of Hannover Hannover Germany; 5 Faculty of Medicine University of Düsseldorf Düsseldorf Germany; 6 National Center for Tumor Diseases German Cancer Research Center (DKFZ) University of Heidelberg Heidelberg Germany; 7 Faculty of Medicine Justus-Liebig-University of Gießen Gießen Germany; 8 Faculty of Medicine University of Regensburg Regensburg Germany; 9 Faculty of Medicine University of Bochum Bochum Germany; 10 Institute for Prevention and Occupational Medicine of the German Social Accident Insurance Institute of the Ruhr-University Bochum (IPA) Bochum Germany; 11 Faculty of Medicine University of Essen Essen Germany; 12 Faculty of Medicine University of Freiburg Freiburg im Breisgau Germany; 13 Department of Psychiatry and Psychotherapy University Hospital of Tuebingen Tübingen Germany; 14 Department of Anaesthesiology and Intensive Care Medicine University Hospital of Cologne Cologne Germany; 15 Department of Otorhinolaryngology Head and Neck Surgery University Hospital of Erlangen Erlangen Germany; 16 Cancer Prevention Unit German Cancer Research Center (DKFZ) Heidelberg Germany; 17 Faculty of Medicine University of Göttingen Göttingen Germany; 18 Institute of Occupational Medicine, Social Medicine and Environmental Medicine Goethe-University of Frankfurt am Main Frankfurt am Main Germany; 19 Division of General Internal Medicine Massachusetts General Hospital Harvard Medical School Boston, MA United States; 20 Cognitive Science and Assessment Institute University of Luxembourg Luxembourg Luxembourg; 21 School of Medicine Federal University of Ouro Preto Ouro Preto Brazil; 22 Health Sciences Centre University of Calgary Calgary, AB Canada

**Keywords:** schools, tobacco prevention, smoking prevention, medical students, medical school

## Abstract

**Background:**

Most smokers start smoking during their early adolescence under the impression that smoking entails positive attributes. Given the addictive nature of cigarettes, however, many of them might end up as long-term smokers and suffering from tobacco-related diseases. To prevent tobacco use among adolescents, the large international medical students’ network Education Against Tobacco (EAT) educates more than 40,000 secondary school students per year in the classroom setting, using evidence-based self-developed apps and strategies.

**Objective:**

This study aimed to evaluate the long-term effectiveness of the school-based EAT intervention in reducing smoking prevalence among seventh-grade students in Germany. Additionally, we aimed to improve the intervention by drawing conclusions from our process evaluation.

**Methods:**

We conduct a cluster-randomized controlled trial with measurements at baseline and 9, 16, and 24 months postintervention via paper-and-pencil questionnaires administered by teachers. The study groups consist of randomized schools receiving the 2016 EAT curriculum and control schools with comparable baseline data (no intervention). The primary outcome is the difference of change in smoking prevalence between the intervention and control groups at the 24-month follow-up. Secondary outcomes are between-group differences of changes in smoking-related attitudes and the number of new smokers, quitters, and never-smokers.

**Results:**

A total of 11,268 students of both sexes, with an average age of 12.32 years, in seventh grade of 144 secondary schools in Germany were included at baseline. The prevalence of cigarette smoking in our sample was 2.6%. The process evaluation surveys were filled out by 324 medical student volunteers, 63 medical student supervisors, 4896 students, and 141 teachers.

**Conclusions:**

The EAT cluster randomized trial is the largest school-based tobacco-prevention study in Germany conducted to date. Its results will provide important insights with regards to the effectiveness of medical student–delivered smoking prevention programs at school.

**International Registered Report Identifier (IRRID):**

DERR1-10.2196/13508

## Introduction

### Background

Most smokers start smoking during their early adolescence with the idea that smoking entails positive attributes; at this age, the health risks of smoking such as those related to vascular disease, lung cancer, and chronic pulmonary disease are too far in the future for them to fathom. Given the addictive nature of cigarettes, however, many smokers might end up as long-term smokers and suffering from severe and potentially deadly tobacco-related diseases [1].

Despite the fact that effectiveness of inpatient smoking cessation was demonstrated in major trials [2] and that these measures were implemented in the guidelines of almost all medical specialties [3], research has shown that physicians in Germany lack both motivation (eg, role incongruence as a major barrier [4,5]) and education to deliver such measures [4-7], especially before the onset of chronic disease [5]. The issue of undertreatment of tobacco use by physicians is known on a global scale [8,9]. It is estimated that global tobacco-attributable mortality will double from 5 million (2010) to 10 million per year in a few decades [1].

Education Against Tobacco (EAT) is a multinational network of medical students that aims to provide science-based tobacco prevention to a large number of adolescents and to thereby sensitize prospective physicians toward the importance of inpatient smoking cessation [10-12]. The network currently involves about 80 medical schools in 12 countries, with 3500 medical students educating more than 40,000 secondary school students in the classroom setting per year using and optimizing self-developed apps and strategies (Multimedia Appendix 1) [13-15]. The two free science-based quit apps of EAT (“Smokerface” and “Smokerstop”) were downloaded more than 400,000 times and translated in the most spoken languages worldwide (Multimedia Appendix 1) [14,15].

The 2018 KiGGS report by the German Robert Koch Institute revealed that 9.3% of German boys and 8.9% of German girls aged 14-17 years smoke cigarettes at least once a week [16]. In spite of the decline in adolescent smoking over the last two decades, prevalence in Germany is among the highest in Europe, and strong socioeconomic differences in smoking habits exist [17-19].

### Current Knowledge on School-Based Tobacco Prevention

Most school-based smoking prevention–related curricula are ineffective, and the evaluation of new curricula is warranted [20]. A recently published evaluation of a short student and student-parent smoking prevention program in Germany did not show significant effectiveness among seventh-grade students (7.6% and 7% prevalence in intervention groups, respectively, vs 10.1% in the control group) at the 24-month follow-up. However, this might have been due to a very low sample size: Only 47 schools were randomized because of an underestimated intracluster correlation coefficient [21,22]. The largest tobacco-prevention program for secondary schools in Germany—the smoke-free class competition—has demonstrated limited effectiveness in making students quit and increasing knowledge among students and was not able to prevent smoking onset [23-25].

Physician-based programs relying on fear-inducing statements show no overall long-term effectiveness in reducing the prevalence of smoking [26-29]. Limited new evidence suggest that asking questions about health consequences rather than making statements might be more effective to at least motivate current smokers to quit [30].

A physician-based multimodal program in Berlin, where students attended a 2-h interactive presentation of smoking-related health consequences, evaluated in a quasi-experimental study suggested significant short-term effects of preventing smoking onset, which might be a promising alternative to the traditional fear approaches of physician-based programs [31]. Outside of schools, a systematic review on inpatient physician-based smoking prevention and cessation for adolescents revealed that behavioral interventions show overall effectiveness in primary care [32].

### Previous Research on Education Against Tobacco

The effectiveness of an earlier version of the EAT curriculum on reducing smoking prevalence among adolescents has only been investigated with a quasi-experimental design (n=1474) with potential sources of bias [10,11]. However, the study showed a significant association of the intervention with lower smoking prevalence among secondary school students in Germany at 6 months of follow-up by motivating them to quit. After this first evaluation, the curriculum was optimized for students with a lower educational level by using cognitive interviewing, as the intervention was found to be less effective in this subgroup. The curriculum received more age-appropriate content, was optimized to be more interactive and gain framed [33], and was equipped with app-based strategies [10,14].

### Education Against Tobacco Apps: “Smokerstop” and “Smokerface”

Photoaging desktop programs in which an image is altered to predict future appearance were effective in motivating girls aged 14-18 years to quit smoking and increased the quit rate in young adults aged 18-30 years of both genders by 21% [34,35]. We took advantage of the broad availability of smartphones and adolescents' interest in appearance to create a free 3D-photoaging smartphone app “Smokerface” [15], which animates the users’ selfies and reacts to touch (Multimedia Appendix 2). It is downloaded 200 times per day, and the current version of the app has a rating of 4.2/5 in the Google Play Store (Google LLC, Mountain View, CA) and 4.5/5 in the Apple AppStore (Apple Inc, Cupertino, CA).

Our second free quit app is called “Smokerstop” and was developed based on theory [36] and evidence [37] from conventional smoking-cessation programs. The underlying concept is the PRIME Theory, which has been described in great detail elsewhere [38,39]. Our app takes into account recent research on adequate coping strategies for craving [40,41]. About 1000 smokers per day use this app to support their quit attempt, and it has an average rating of 4.5/5 in the AppStore and Play Store. Smokerface motivates people to remain smoke free or to make a quit attempt and is likely to help with continuous abstinence [14]; in contrast, Smokerstop supports quitters who are already prepared to set a quit date. Both apps are a part of our school-based intervention.

We designed this randomized trial to answer the following questions:

- Does medical student–delivered prevention by EAT show effectiveness in reducing smoking prevalence in secondary schools?
- Which subgroups (ie, gender, education level, and cultural background) benefit most from this intervention?
- Is this low-cost campaign effective in convincing students to use the apps?
- Which students are more likely to use an app revealing the photoaging effects of smoking?

## Methods

### Ethics Approval

The study protocol was approved by the ethics committee of the University of Giessen and the ministries of cultural affairs of the five participating federal states. Written informed consent was obtained by the responsible teachers from both the participants themselves and their parents. All participant information will be stored in locked file cabinets in areas with limited access. Participants’ personal information will not be released outside of the study without written permission of the participants. Study results will be disseminated at national and international conferences, in peer-reviewed journals, on our websites, and throughout the multinational EAT network.

### Trial Design

A randomized controlled multicentered trial with two parallel groups is underway (ClinicalTrials.gov: NCT02697409). A total of 13 German EAT groups are participating, each functioning as a study center. The primary outcome is the between-group difference in smoking prevalence from baseline to follow-up. Randomization was externally and centrally performed via a computer on a school level with a 1:1 allocation.

A total of 144 secondary schools in five federal states of Germany participated in the baseline survey in the first half of the school year (September 2016 to April 2017, depending on the federal state) prior to randomization. The randomization of schools based on the baseline data was performed from November 2016 through May 2017 by the Coordination Center for Clinical Studies Marburg (KKS Marburg) as a blocked randomization combined with stratified randomization by study center and smoking prevalence, in order to ensure a balance of participant characteristics in each group. Immediately after randomization, schools were informed of their group allocation (intervention or control) and appointments were made for the implementation of the EAT curriculum in the intervention group. To assess the quality of the intervention, we implemented a process evaluation including four points of view: medical student volunteers and training supervisors after training via the EAT curriculum as well as teachers and students within 24 hours postintervention. The first follow-up survey was conducted 9 months after the intervention. The second follow-up was conducted at 16 months (April 2018 to February 2019), and the third follow-up will be conducted at 2 years (December 2018 to October 2019). In order to assure comparability between the two groups, we calculated the average number of days between randomization and intervention in each study center for the intervention group and added these numbers to the randomization date of the schools in the control group when assessing the dates for the follow-up surveys.

### Intervention

#### Before the Visit

We sent letters to teachers to prepare them for our visit. Parents received letters to motivate them to quit smoking via the Smokerface App and to attempt to quit with the Smokerstop App [42] in case they are smokers themselves while informing them on how to best ensure that their children do not take up the behavior, summing up recent pertinent scientific publications in layman’s terms [43-46]. The students were advised to prepare for our intervention by downloading the Smokerface App on their smartphone [14]. The medical student volunteers were trained in the 2016 EAT curriculum by experienced supervisors in all cases and by long-term group leaders of the EAT network of medical students, via a standardized preparation curriculum.

#### In Schools

In the first part of the intervention, lasting for about 45 minutes, all participating classes of Grade 7 will gather in a large room under the supervision of at least two medical students. For the first 30 minutes, students will be interactively involved in a PowerPoint (Microsoft Corp, Redmond, WA) presentation that discusses how smoking affects the performance of German soccer players, addiction, costs, relaxation/happiness, and strategies of the tobacco industry and are interviewed about how they would advertise cigarettes to the rest of their grade. In the last 15 minutes, our photoaging app is implemented into the school setting via a self-developed strategy called “mirroring”: The students’ altered 3D self-portraits on mobile phones or tablets are “mirrored” via a projector in front of their whole grade [14]. In a recently published pilot study, we were able to demonstrate that this type of implementation influences multiple predictors of smoking in accordance with the theory of planned behavior [14,47].

The second part of the intervention, lasting about 90 minutes, is designed to be as interactive as possible: The students are sent to their classrooms where they are split into three groups with three medical students per room. There, they rotate to four different stations in the classroom, which discuss age-appropriate information, ask about their own experiences, and have them conduct their own experiments.

### Different Tobacco Products and Extraction of Substances of Tobacco Smoke

In the first part, different products (including electronic cigarettes [e-cigarettes], waterpipe, and cigarettes) are displayed and explained, and their harmfulness is discussed in a gain-framed manner.

In the second part, the students will observe an experiment using a napkin, a prepared plastic bottle filled with water, and a cigarette. The cigarette is fixated at the bottleneck via a rubber plug and burned, while the water is drained through a hole in the bottom of the bottle to create a vacuum. After the vacuum makes the smoke flow into the bottle, the cigarette is removed, and the napkin is put around the bottleneck. The smoke then gets blown out of the bottle through the napkin, which demonstrates the tar in the smoke by the discoloration of the napkin. When proper ventilation is not ensured, the medical students and school students will conduct the experiment outside to avoid unnecessary exposure to second-hand smoke.

### Attractiveness and Mechanisms Related to the Face

In the first part, pictures of monozygotic smoking/nonsmoking twins are displayed, which are extracted from the publication of Okada et al [48]. The students are asked which twin is the smoker and what differences they note between the twins.

**Figure 1:**
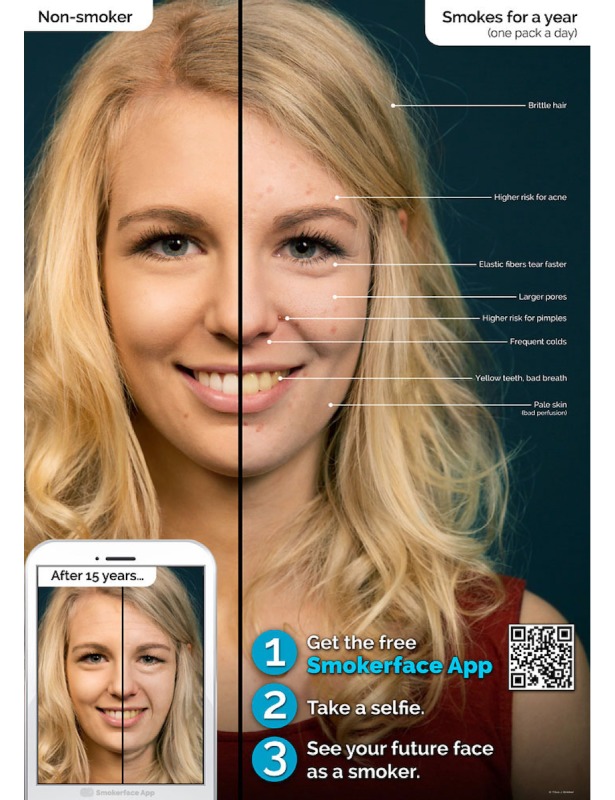
Female poster at baseline.

**Figure 2:**
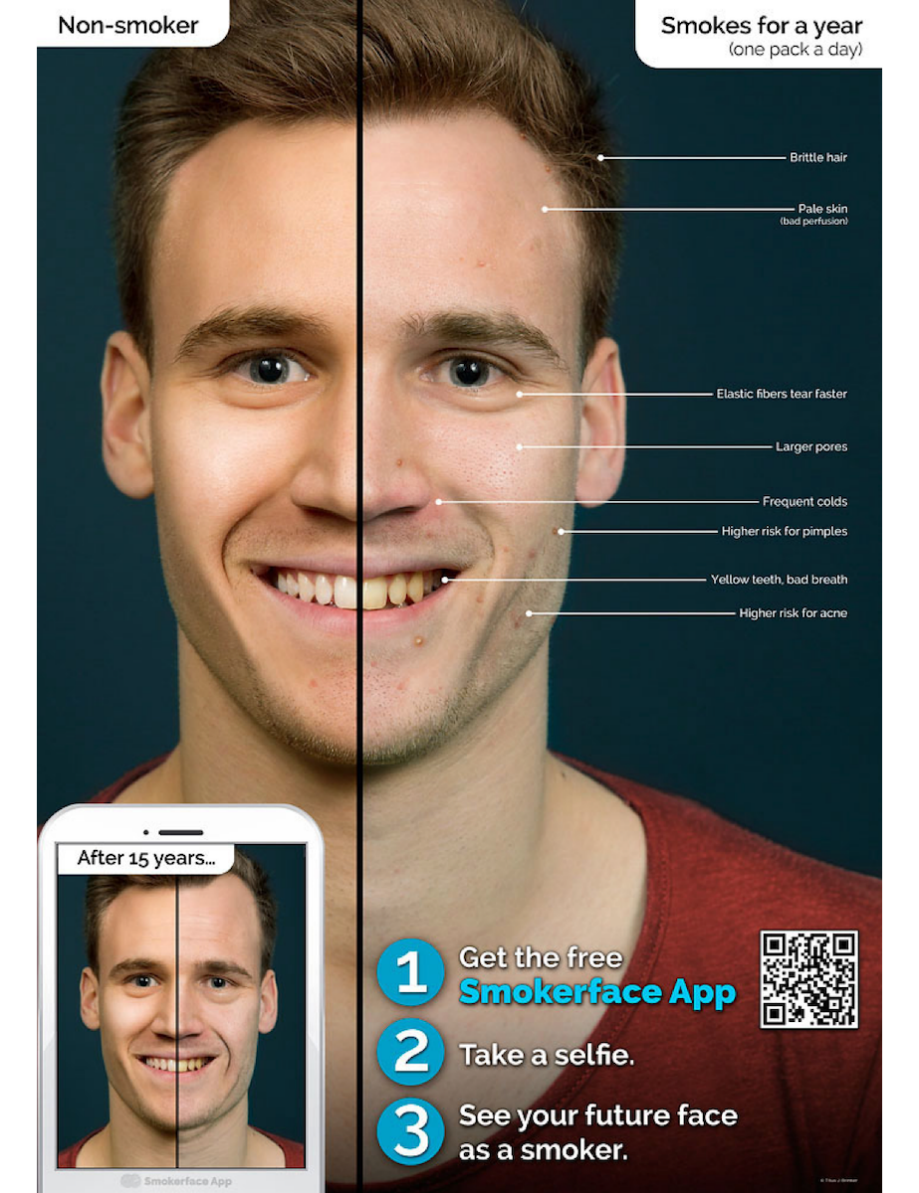
Male poster at baseline.

**Figure 3:**
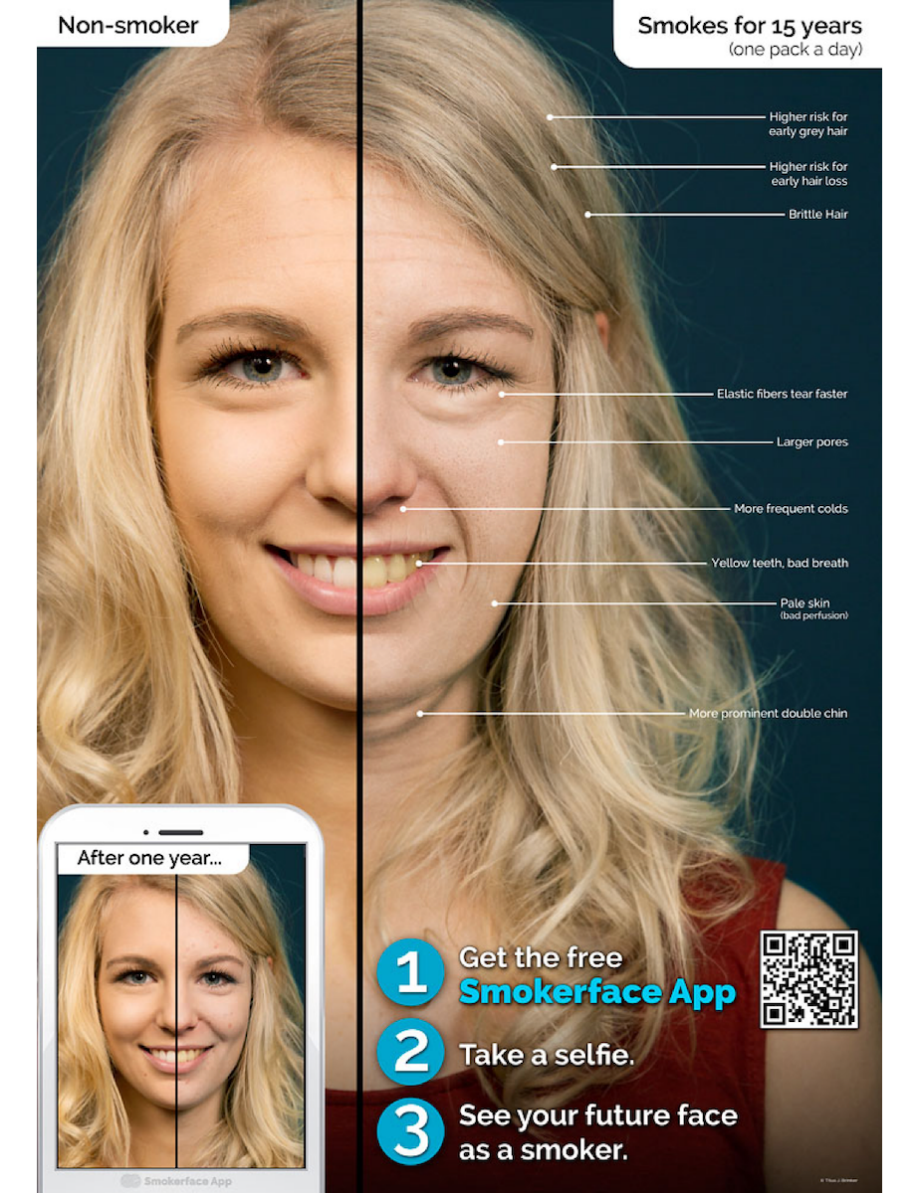
Female version of post-15 year Smokerface poster at 1 year postintervention.

**Figure 4:**
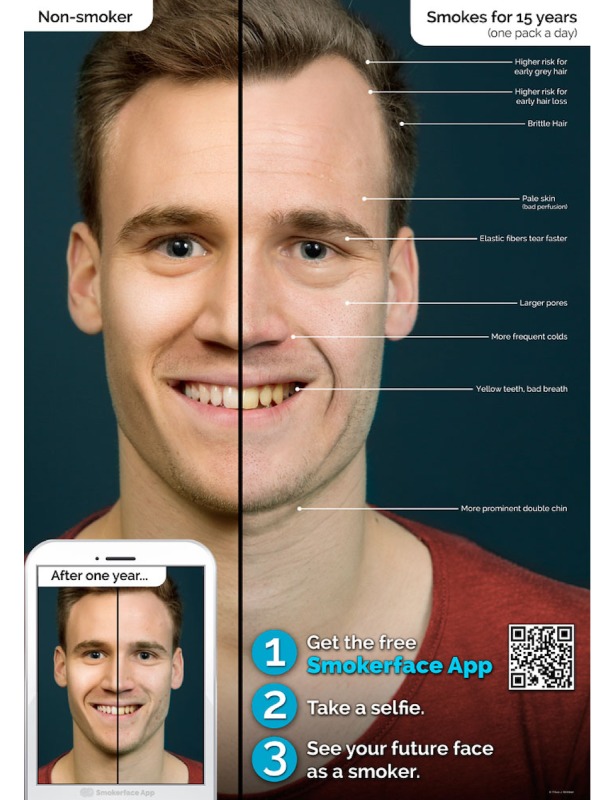
Male version of post-15 year Smokerface poster at 1 year postintervention.

In the second part, Galaxy Tab A tablets (Samsung Electronics Inc, Seoul, Korea) are used to show each student the effects of smoking/nonsmoking on their own faces by the help of the photoaging app Smokerface that we described and piloted in great detail elsewhere [14,15]. As such, the students’ faces are captured via a selfie and photoaged into a 1- to 15-year older version of themselves (normal aging vs normal aging + smoking) with animated touch effects (Figures 1-4,Multimedia Appendix 1). This intervention has been shown to influence numerous predictors of smoking in students of this age group in accordance with the theory of planned behavior and as demonstrated in our recent paper [14].

### Performance Benefits of Nonsmoking

Performance benefits of nonsmoking (physical performance, stress, and common colds) and understanding the *mechanisms* of *how* tobacco smoking affects the body with age-appropriate examples (eg, occluded vessels lead to loss of connective tissue in women’s breasts, which is equivalent to less volume/tightness; impotence in both men and women; pale skin; and mechanisms of acne); this is explained via pencil and paper drafts and interactive questions. In addition, obesity [49,50], lung growth [51], and body growth impairment in adolescent smokers are discussed using a body model, paper-pencil sketches, and growth curves [50].

### Personal Experiences: How Can I Stay Away From Smoking?

The aim of this station is to discuss the students’ own experiences with tobacco and how they reacted to peer pressure in the past. The group’s knowledge/experience is shared and discussed in a team setting where the medical students take the role of older friends to complement the students’ experiences with their own experiences in order to increase students’ perceived self-efficacy, which is the most important predictor of future smoking according to the theory of planned behavior [47]. It has been shown to predict both the intention to smoke and actual smoking behavior in a meta-analysis [52].

At the end of the classroom seminar, we will ask for the students’ final judgments on smoking to create positive peer pressure and influence students’ subjective norm in accordance with the theory of planned behavior [47]. Health consequences are not discussed in great detail, as fear approaches were proven to be ineffective and information on smoking-related diseases can be found on every cigarette pack [25]. As a final exercise, all students breathe through a straw after having physically exercised in the classroom together to learn how lung impairment due to smoking feels with exercising. The medical students hang up the first two self-developed posters of the Smokerface App poster campaign, which has been described in great detail elsewhere [53].

#### After the Visit

One-year postintervention, the Smokerface App posters showing a 1-year difference of smoking are replaced with a version showing a 15-year difference.

According to a recent Cochrane review [20], smoking parents should be involved and encouraged to stop smoking, as adolescents are twice as likely to start smoking if their parents do [42]. However, increased perceived parental control increases the likelihood of adolescents to choose smoking friends and needs to be avoided [43]. To further increase the use of the apps and to guide smoking cessation among parents within the intervention group, the intervention posters will be complemented by letters to the students and parents (delivered along with the questionnaires at the 9-, 16-, and 24-month follow-ups).

#### School Involvement

Schools in the intervention group are offered a long-term partnership with their local EAT group, where students also deliver the EAT curriculum to Grade 7 students of the following school years. Inviting the students back is not mandatory. Students taking part in the study do not get a second intervention.

### Monitoring

#### App Stability

The stability of the Smokerface App will be monitored during the study period via the Crashlytics app (San Francisco, CA).

#### External Data Monitoring Committee

As suggested by the Standard Protocol Items: Recommendations for Interventional Trials guidelines, all primary analyses will be performed externally and the raw datasets will be sent to the Collaboration Center for Clinical Trials in Marburg, Germany, for external monitoring [54].

### Participants

Students from Germany attending Grade 7 in all types of regular secondary schools in Germany were eligible. Schools of other types such as special schools for mentally handicapped children or Rudolf Steiner schools, schools in other countries, or schools that had previously participated in an EAT event were not eligible. Schools were contacted by each study center individually, and therefore, only schools in the vicinity of the participating medical schools could enter the study.

### Procedure

Data at baseline and follow-up are collected via a published questionnaire developed for and used in our previous investigation in the same age group [53]. All items were based on three established studies declared as high quality by the recent Cochrane review [20] and were either used in their original form or adapted to the specific circumstances of the recent study [55-57]. Data for process evaluation was collected via a newly devised questionnaire asking for feedback on the curriculum, the medical students, and the Smokerface app specifically. Most items were assessed using a 4-point Likert scale.

### Data Collection

Teachers will collect the data and hand out a modified protocol, used in the Hutchinson Smoking Prevention Project, which was provided by and discussed with the authors as well as used in our previous investigation [55].

### Randomization

Schools were externally and centrally randomly allocated to the control or intervention group by the KKS in Marburg, Germany. This center used permuted-block randomization via a computer with random block sizes. Stratification by a predefined smoking prevalence (≥2.65% or <2.65%) at baseline was used to balance group allocation. Schools were allocated to the control or intervention group in a ratio of 1:1 (except for Bonn, 2:1). A total of 72 schools were randomized into the control group and intervention group.

### Outcomes

The primary outcome is the between-group difference of the change in smoking prevalence from baseline to the 24-month follow-up. Secondary outcomes are between-group differences in change in smoking-related attitudes in accordance with the theory of planned behavior and the number of new smokers, quitters, and never-smokers after 24 months. For all outcomes, the number needed to treat will be calculated. Considering the short nature of the intervention, we predefined a number needed to treat below 50 as clinically relevant. Students are defined as smokers if they report having used cigarettes, at least one day in the 30 days preceding the survey, in accordance with the established National Youth Tobacco Survey definition [58]. Students who report not having smoked cigarettes in the past 30 days were defined as nonsmokers. All participants who report having smoked more than a puff in the past (beyond the past 30 days) were defined as ex-smokers.

### Statistical Considerations

#### Sample Size Calculation

The sample size for the primary outcome was calculated with a two-sided Chi-square test and multiplied by the correction factor design effect (design effect=1 + [cluster size−1]* intraclass correlation coefficient) to adjust for correlation with regard to smoking prevalence within a cluster. We calculated an intraclass correlation coefficient of 0.033, based on the data from our recently published study on smoking behavior in Germany (analysis of variance estimator by Zou and Donner) [11,59].

To detect a between-group difference of 3% change in smoking prevalence from baseline to 24 months of follow-up with an alpha of 5% and a test power of 70%, we calculated a sample size of 5645 to 15,715 participants, depending on the difference in smoking prevalence between the two groups (2% vs 5% up to 9% vs 12%, respectively) and with an assumed dropout rate of 30% during the follow-up. Assuming an average cluster size of 100 participants, approximately 56-157 schools needed to be randomized. The dropout rate of 30% is appropriate for our 24 months of follow-up, as we observed less than 20% dropout in our recent 6-month investigations [10,11].

#### Data Entry

Data entry will be performed using the current software version of Formic Fusion by the Xerox AG (Kloten, Switzerland) and recommended scanners provided by the Interdisciplinary Centre for Educational Research at the University of Duisburg-Essen.

#### Analysis

To examine baseline differences in students’ characteristics in our experimental design, we will use Chi-square tests for the categorical variables and *t* tests for the continuous variables. To test for differences in baseline and follow-up smoking prevalence between groups, we will use a cluster-adjusted Mantel-Haenszel Chi-square test [60] at a two-sided significance level of 5%. In the main analysis, hierarchical linear models (HLM) will be applied. HLM can handle the nested structure of the data and will be used to test for between-group differences in within-group changes in smoking behavior over time. HLM will also be used to investigate the influence of further covariates (such as gender, cultural background, and social characteristics) and time-dependent behavior in secondary analyses. Statistical analyses will be performed using the newest version of SPSS Statistics (IBM Corp, Armonk, New York).

The potential effects of missing data on the results will be assessed via sensitivity analysis. For this, dropouts (ie, participants who withdraw consent for continued follow-up or who are missing in the classroom during the survey) will be included in the analysis by applying multiple imputations [61]. 

## Results

### Baseline Characteristics

Overall, 11,286 students participated in the baseline survey (Table 1). The mean age was 12.32 years (range 9-17) and 50% (5490/10,965) were female. Of the total, 47.2% (68/144) of the schools were grammar schools, which provide general qualification for university entrance at the end; the rest were comprehensive schools, which provide a general certificate of secondary education at the end. At baseline, 2.6% of participants reported smoking within the last 30 days, while 84.5% (9296/11,002) reported never having smoked a cigarette (never-smokers). Current smokers reported having smoked an average of 58.92 cigarettes (SD 158.38) within the last 30 days, amounting to two cigarettes per day on an average. In addition, 4.7% of participants reported having smoked an e-cigarette within the last 30 days, with an average of 6.19 days of use. Tobacco waterpipe smoking was reported by 3.0% of the participants, with an average of 7.30 days of use. Further, 2.2% of participants self-reported using a steam stone waterpipe and cigar/cigarillo (0.6%), chewing tobacco (0.2%), marijuana (1.2%), and other nonspecified tobacco products (0.5%). The survey also identified 3.6% of participants as users of at least two tobacco products. Moreover, 38.5% of participating students reported having at least one smoking parent, 11.2% identified one of their best friends as a smoker, and 11.7% identified an older sibling as a smoker.

### Process Evaluation

Our process evaluation is quite extensive, and most of these data are too detailed for publication but help with internal monitoring. The full process-evaluation analysis is provided in Multimedia Appendix 3; only the core parameters are presented in the manuscript.

The process-evaluation surveys were filled out by 324 medical student volunteers (“mentors”), 63 medical student supervisors (“educators”), 4896 students, and 141 teachers. In 59 of the 72 schools in the intervention group, we were allowed to survey the students after the intervention. On an average, 29.5 mentors were educated per medical school by 6.3 educators at the 13 medical schools involved in the study. With an average age of 21.8 years, mentors were about 1 year younger than the average educator (age, 22.7 years), which is reflected by the fact that only 44.1% (142/322) of mentors, as opposed to 82.5% (52/63) of educators, were in the clinical phase of medical school (Table 2).

We received mentor questionnaires from 11 of the 13 medical schools (all except Heidelberg and Düsseldorf) and educator feedback from 10 of the 13 medical schools (all except Giessen, Heidelberg, and Cologne; Table 3). All volunteering medical students for the study received training.

**Table 1:** Baseline characteristics.

Characteristics			Total	Intervention group	Control group
Students, n (%)			11,286 (100)	5732 (50.8)	5554 (49.2)
Schools, n (%)			144 (100)	72 (50.0)	72 (50.0)
Grammar schools, n (%)			68 (47.2)	36 (50.0)	32 (44.4)
**Gender, n (%)**			**10,965 (97.2)**	**5584 (97.4)**	**5381 (96.9)**
	Female		5490 (48.6)	2777 (49.7)	2713 (50.4)
	Male		5475 (48.5)	2807 (50.3)	2668 (49.6)
Age, n (%)			11,054 (97.9)	5624 (50.9), mean 12.32, median 12 (SD 0.67), range 9-17	5430 (49.1), mean 12.33, median 12, (SD 0.64), range 9-17
**Current cigarette smoking (at least once in past 30 days), n (%)**			**285/11,127^a^ (2.6)**	**137/5669^a^ (2.4)**	**148/5458^a^ (2.7)**
	Average number of cigarettes smoked in past 30 days per current smoker (SD)		58.92 (158.38)	56.71 (137.88)	61.013 (176.13)
**Average days of use in the past 30 days per current smoker (SD)**			**8.68 (10.81)**	**9.14 (11.02)**	**8.25 (10.63)**
	1-2 days, n (%), average number of cigarettes per day		145 (1.4), 0.89	63 (1.2), 0.95	82 (1.6), 0.85
	3-5 days, n (%), average number of cigarettes per day		30 (0.3), 3.13	18 (0.3), 4.06	1.75 (0.2), 1.75
	6-9 days, n (%), average number of cigarettes per day		19 (0.2), 2.45	13 (0.2), 2.27	6 (0.1), 2.83
	10-19 days, n (%), average number of cigarettes per day		26 (0.2), 3.33	11 (0.2), 5.18	15 (0.3), 1.97
	20-29 days, n (%), average number of cigarettes per day		10 (0.1), 3.90	5 (0.1), 2.50	5 (0.1), 5.30
	All 30 days, n (%), average number of cigarettes per day		43 (0.4), 10.12	23 (0.4), 8.52	20 (0.4), 11.95
	Not smoked in the past 30 days (nonsmokers), n (%)		10,842 (97.4)	5532 (97.6)	5310 (97.3)
	Never tried smoking, not even a puff, n (%)		9458/11,074^a^ (85.4%)	4835 (85.7)	4623 (85.1)
	Never smoked a cigarette (never-smokers), n (%)		9296/11,002^a^ (84.5%)	4754 (85.0)	4542 (84.0)
**Ex-smokers who smoked... n (%)**					
	More than once per week		122 (1.1%)	57 (1.0%)	65 (1.2%)
	Less than once per week		122 (1.1%)	66 (1.2%)	56 (1.0%)
**Average age of first puff (years), n (%)**					
	≥8		364 (22.8)	161 (20.5)	203 (25.0)
	9-10		238 (14.9)	124 (15.8)	114 (14.0)
	11-12		780 (48.9)	389 (49.6)	391 (48.2)
	13-14		214 (13.4)	110 (14.0)	104 (12.8)
	Intention to smoke cigarettes^b^		0.44	0.45	0.44
	Do you intend to quit cigarettes?^c^		0.40	0.43	0.36
	Current tobacco waterpipe smoking, n (%), mean days of use in the past 30 days (SD)		330 (3.0), 7.30 (9.36)	163 (2.9), 7.83 (9.84)	167 (3.1), 6.77 (8.87)
	Current e-cigarette smoking, n (%), mean days of use in the past 30 days (SD)		519 (4.7), 6.19 (8.60)	250 (4.4), 6.44 (8.63)	269 (5.0), 5.96 (8.57)
	Current cigar or cigarillo smoking, n (%), mean days of use in the past 30 days (SD)		72 (0.6), 9.37 (11.87)	30 (0.5), 8.27 (11.52)	42 (0.8), 10.15 (12.19)
	Current chewing of tobacco, n (%), mean days of use in the past 30 days (SD)		25 (0.2), 14.90 (12.88)	11 (0.2), 16.32 (13.77)	14 (0.3), 13.79 (12.54)
	Current use of marijuana, n (%), mean days of use in the past 30 days (SD)		128 (1.2), 12.33 (12.68)	64 (1.1), 10.68 (11.97)	64 (1.2), 13.98 (13.25)
	Current use of steam stone waterpipe, n (%), mean days of use in the past 30 days (SD)		247 (2.2), 6.32 (8.65)	117 (2.1), 6.07 (8.43)	130 (2.4), 6.54 (8.87)
	Current use of other tobacco product, n (%), mean days of use in the past 30 days (SD)		51 (0.5), 9.21 (11.60)	23 (0.4), 10.02 (12.54)	28 (0.5), 8.54 (10.94)
	Current use of *at least* two tobacco products, n (%)		402 (3.6)	189 (3.3)	213 (3.9)
	Current use of electronic cigarettes and cigarettes, n (%)		145 (1.3)	67 (1.2)	78 (1.4)
	Current use of waterpipe with tobacco and cigarettes, n (%)		99 (0.9)	52 (0.9)	47 (0.9)
**Smoking in a social environment, n (%)**					
	I have at least one smoking parent		4278 (38.5)	2188 (38.6)	2090 (38.4)
	One of my best friends smokes		1174 (11.2)	567 (10.6)	607 (11.8)
	I have an older sibling that smokes		1252 (11.7)	623 (11.5)	629 (12.0)
**Migration/socioeconomic background, n (%)**					
	Both parents born in Germany		6740 (62.6)	3467 (63.2)	3273 (62.1)
	One parent born in Germany		1724 (16.0)	877 (16.0)	847 (16.1)
	No parent born in Germany		2296 (21.3)	1146 (20.9)	1150 (21.8)
	School performance (self-reported point average), n (%), mean (SD)		10,757 (95.3), 2.42 (0.85)	5475 (50.9), 2.40 (0.84)	5282 (49.1), 2.43 (0.86)
**Education level of parents^d^** **, score**					
	Father		3.90	3.90	3.91
	Mother		3.84	3.84	3.84
**“Do you live in the same household with your parents?”, n (%)**					
	I live with both parents		8430 (76.5)	4320 (77.0)	4110 (75.9)
	With mother but not father		1964 (17.8)	994 (17.7)	970 (17.9)
	With father but not mother		274 (2.5)	131 (2.3)	143 (2.6)
	Neither mother nor father		358 (3.2)	167 (3.0)	191 (3.5)
**Survey quality, n (%)**					
	“Anonymity was explained to me before I filled out the questionnaire.”		10,286 (94.0)	5242 (94.1)	5044 (93.9)
	“It was made clear that nobody knows that I filled out this questionnaire.”		8349 (76.6)	4229 (76.1)	4120 (77.0)

^a^These are valid answers from the questionnaire.

^b^Scale 0-6 (0=I am very sure that I will never smoke to 6=I believe that I will start smoking within the next month).

^c^Scale: 0-3 (0=no to 3=within the next month).

^d^Score: 1-5 (1=not completed school education to 5=completed university).

**Table 2:** Participant characteristics^a^.

Variable		Mentors receive education for classroom visit (n=324)	Educators deliver education to mentors (n=63)
Number of mentors/educators per medical school, mean (SD)		29.5 (16.7)	6.3 (3.4)
**Age (years), mean/N (SD); median (range)**		**21.8/320 (2.8); 21 (18-32)**	**22.7/63 (1.5); 23 (20-28)**
	Female, n/N (%)	217/322 (67.4)	37/61 (60.7)
	Male, n/N (%)	101/322 (31.4)	24/61 (39.3)
Preclinical phase of medical school, n/N (%)		180/322 (55.9)	11/63 (17.5)
Clinical phase of medical school, n/N (%)		142/322 (44.1)	52/63 (82.5)
Nonsmokers, n/N (%)		294/322 (91.3)	56/57 (98.2)
Ex-smokers, n/N (%)		24/322 (7.5)	0/63 (0)
Smokers, n/N (%)		4/322 (1.2)	1/57 (1.8)
At least one parent not born in Germany, n/N (%)		152/321 (47.4)	18/62 (29.0)

^a^The denominator for all percentage values is the number of valid cases (number of questionnaires with valid answers).

**Table 3:** Number of mentors and schools.

Medical school	Number of educated mentors, n/N (%)	Number of visited schools, n/N (%)
Bochum	38/324 (11.7)	5/72 (6.9)
Bonn	36/324 (11.1)	6/72 (8.3)
Düsseldorf	—^a^	11/72 (15.3)
Erlangen	20/324 (6.2)	11/72 (15.3)
Essen	20/324 (6.2)	5/72 (6.9)
Freiburg	40/324 (12.3)	4/72 (5.6)
Hannover	12/324 (3.7)	5/72 (6.9)
Köln	3/324 (0.9)	4/72 (5.6)
Gießen	50/324 (15.4)	5/72 (6.9)
Göttingen	11/324 (3.4)	1/72 (1.4)
Regensburg	48/324 (14.8)	4/72 (5.6)
Tübingen	46/324 (14.2)	5/72 (6.9)
Heidelberg	—^a^	6/72 (8.3)

^a^No questionnaires from mentors were handed in.

When asked about their perception of the training, 99.7% (318/319) of mentors and 100% (63/63) of educators responded positively to whether “overall, the training made sense” for the mentors. A total of 96.6% (311/322) of mentors and 100% (63/63) of educators agreed to the statement, “I feel well prepared,” although only 70.6% (228/323) of the mentors agreed that they were able to train their didactic skills. Furthermore, 94.4% (305/323) of the mentors and 100% (61/61) of educators answered with “fully correct” or “rather correct” to the statement, “It increased my motivation to advise my future patients not to smoke” (Table 4).

General feedback on the curriculum was gathered in surveys for all four viewpoints (Multimedia Appendix 3). Here, 90.6% (4361/4814) of students, 93.9% (123/131) of teachers, 98.4% (311/316) of mentors, and 95.2% (60/63) of educators answered positively to the statement that the intervention “will motivate them (the students) to be non-smokers.” Feedback on the medical students was also very positive, with 95.9% (4642/4819) of students and 97.8% (135/138) of teachers answering positively to the statement, “overall, they (the medical students) left a very good impression.”

**Table 4:** Participant perceptions. Used scale: 1=fully correct, 2=rather correct, 3=rather not correct, 4=not correct at all.

Variable		Mentors receive education for classroom visit		Educators deliver education to mentors	
		Mean (SD)	Percentage base of valid cases (%^a^)	Mean (SD)	Percentage base of valid cases (%)
**What influence did the education/training have on yourself?**		
	Increased my motivation not to smoke	1.5 (0.8)	320 (92.2)	1.5 (1)	63 (85.7)
	I learned new things about tobacco as a topic	1.8 (0.9)	324 (76.2)	1.8 (0.9)	63 (73.0)
	It increased my awareness about the harms of tobacco	1.8 (0.8)	323 (84.8)	1.8 (1)	61 (85.2)
	It increased my motivation to advise my future patients not to smoke	1.3 (0.6)	323 (94.4)	1.2 (0.4)	61 (100)
**How did you perceive the training?**	
	It was fun	1.3 (0.5)	324 (99.1)	1.2 (0.4)	63 (100)
	It was interesting	1.3 (0.5)	324 (98.1)	1.3 (0.5)	63 (98.4)
	I feel well prepared	1.5 (0.6)	322 (96.6)	1.3 (0.5)	63 (100)
	I was able to train my didactic skills	2 (0.9)	323 (70.6)	1.4 (0.5)	63 (98.4)
**Global feedback**	
	Overall, the training made sense	1.2 (0.4)	319 (99.7)	1.2 (0.4)	63 (100)
	I would recommend EAT to other medical students	1.1 (0.4)	315 (99.7)	1.1 (0.3)	63 (98.4)

^a^Percent of top two (1 or 2) related to valid cases.

## Discussion

### Overview

This is the first major randomized trial on a medical student–delivered smoking prevention program in the school setting. Our network previously investigated an early version of the EAT curriculum in a quasi-experimental prospective evaluation with a 6-month follow-up (n=1474) as well as the 2014 EAT curriculum in a smaller randomized controlled trial (n=1504) with a 12-month follow-up and a high loss to follow-up [62]. Chances and synergy effects of a medical student intervention are in need of further evaluation from all angles. The investigated intervention is available in the area around the 13 participating medical schools. The number of schools able to receive this intervention is limited by the capacity of the local EAT group.

### Baseline Characteristics

Our baseline survey includes the major predictors of adolescent smoking, as described in the literature [56,57]. The distributions of relevant characteristics over the two groups are balanced, indicating successful randomization. For example, the students in the intervention and control groups are similar with regard to the current smoking prevalence (2.4% and 2.7%, respectively), never-smoking prevalence (85.0% and 84.0%, respectively), and the proportion of those having at least one smoking parent (38.6% and 38.4%, respectively).This large study is conducted in five German federal states. Our definitions for the smoking status of the various monitored tobacco products stem from the National Youth Tobacco Survey by the Center for Disease Control (Atlanta, United States) [57]. Teachers are used as data collectors and were handed out a modified protocol, as used in the Hutchinson Smoking Prevention Project to ensure international comparability.

This is also the first national study to show that current e-cigarette prevalence is higher than cigarette smoking prevalence in Grade 7 students from secondary schools (2.6% use cigarettes and 4.7% use e-cigarettes). More than a quarter of these (1.3% of the total sample) currently use both products at the same time. The epidemiologic data presented here are therefore also valuable, considering that the most cited and most recent surveys in Germany were conducted via telephone interviews, a method showing poor consistency with biochemical validation in our age group [17,63]. We are not using biochemical validation in our study because it would have to take place in the school setting with previous notice on the day the paper questionnaires are given out. This would compromise the comparability of data obtained on that day, since students may answer according to social desirability.

### Quality of Data Collection

We monitored the quality of the data collection with the following two items: (1) Was it explained to you that nobody else than the researchers would see your questionnaire? (2) Anonymity was explained to me before filling out the questionnaire.

A total of 76.6% (8349/10,899) of the students remembered at the end of the questionnaire that the data collectors had explained the confidentiality and 94% (10,286/10,943) of the students stated that anonymity was explained to them.

We were obliged to obtain active consent from the parents and students. Of the students in the schools under investigation, who were registered for the study by their responsible teacher, 83.5% (11,286/13,521) participated in the baseline survey and had obtained parental consent. The teachers are responsible for guaranteeing that only students with parental consent fill out the paper questionnaires; therefore, it is possible that students deliberately or undeliberately failed to present their teachers with a filled out parental consent form and were consequently excluded from data collection.

### Process Evaluation

Our process evaluation captures the view of all four participating parties (educators, mentors, students, and teachers) in the preparation and implementation of the intervention. We did not obtain data on the mentors of two of the larger study centers (Düsseldorf and Heidelberg), which makes it more difficult to draw conclusions regarding perceived proficiency in the curriculum beforehand and outcome measured by student’s impression of the intervention afterward. The educator’s viewpoint is not an individual assessment of each mentor’s proficiency but a group evaluation, since mentors were taught in groups of up to four people. Even though this leaves room for inaccuracy in individual assessment, an educator to mentor ratio of 1:1 would have been too time consuming, considering the 3-hour training. Furthermore, mentor training was intentionally designed for mentors to practice supervising a group of listeners and repeating relevant facts in their own words to promote a finer grasp of what the curriculum is trying to accomplish at every step. Accordingly, 96.6% (311/322) of mentors reported “feeling well prepared” for the intervention. Noticeable findings from the process evaluation were derived from comparison of different viewpoints on the general outlook and specific components of the intervention. A total of 94.3% (296/314) of mentors, 96.8% (61/63) of educators, and 93.2% (124/133) of teachers reported that students learned the benefits of nonsmoking that were new to them. However, only 76.1% (3667/4819) of the students agreed to this statement. Considering that the average mentor was only 21.8 years old and graduated not too long ago, we interpret this finding mainly as a sign of increased awareness of tobacco-related health aspects in the younger generation, possibly because it received increased media coverage over the last few years. It is also possible that students overestimate how profound their knowledge was beforehand. We found similar discrepancies in the specific feedback to our Smokerface app. Although 82% (105/128) of teachers, 85% (272/320) of mentors, and 76.2% (48/63) of educators rated the alterations of people’s selfies to be “realistic,” only 47.3% (2274/4807) of students agreed with this viewpoint. This was especially surprising because at the same station, students were shown pictures of identical twins, one of them being/having been a smoker, during the classroom intervention. When it came to their own face and appearance, students showed reluctance to accept the gravity of skin aging for smokers. When asked which of the presented short-term effects of smoking was most relevant to them, grammar school students reported stunted lung growth most frequently (583/2376, 24.5%), while comprehensive school students most often reported pimples as their primary concern (440/1891, 23.3%). The curriculum should be adapted to cater to these concerns (focus on appearance vs noxious effects), depending on which type of school is receiving the intervention. Only 49.1% (2338/4765) of students made a selfie with the provided tablet during the great hall presentation, even though everyone was supposed to be given the opportunity. Feedback by study centers suggests that time management was an issue: 45 minutes of presentation did not leave much buffer time for delay, so students arriving late or great halls not being prepared by janitors ultimately resulted in several cases of time management issues. A discussion of whether the presentation will be slimmed down or formally extended to 60 minutes will take place in the near future. The short time frame may also be the reason why 89.3% (92/103) of teachers considered the presentation to be “very good” compared to 98.5% (128/130) for the classroom intervention, where time management was not reported to be an issue.

### Generalizability

As this study is conducted only in Germany, the results might not be generalizable to other cultural or national settings. However, the EAT network is quickly expanding to other countries such as Brazil, and research is also conducted there using part of the EAT curriculum [64]. Participating schools are mostly located in urban areas close to larger cities with medical schools. Therefore, the results might not be generalizable to schools in rural areas. However, since medical student–delivered interventions are unlikely to be widely available there, these concerns might be negligible.

Part of the investigated intervention is easy to implement and can be added to existing school-based programs. We provide original posters in high resolution for offset print on our website [65].

### Conclusions

Our research provides a great opportunity to evaluate the curriculum of a multinational medical student network. Involving and engaging medical student volunteers in interactions with young students can sensitize them toward the current trends in and danger of smoking. Our baseline analysis shows good comparability between the groups at baseline after randomization and provides new insights into the prevalence of smoking and the use of e-cigarettes among students in the seventh-grade in Germany. With our process evaluation, we were able to ensure the quality of the intervention as well as the medical student training and receive positive feedback on the curriculum and medical students’ performance. The feedback will help further optimize the intervention with regard to the type of school receiving the intervention and the organizational structure, especially the great hall presentation. We are looking forward to sharing our final report on the follow-up results and changes implemented, as EAT is an ongoing project expanding in size and availability.

## Appendix

Multimedia Appendix 1 Video introduction to Education Against Tobacco.

Multimedia Appendix 2 Animated touch-effect "cough" of the Smokerface App.

Multimedia Appendix 3 Detailed process evaluation.

## Abbreviations

EAT: Education Against Tobacco
e-cigarettes: electronic cigarettes
HLM: hierarchical linear models
