# Supplementary material for: Process Evaluation of a Medical Student–Delivered Smoking Prevention Program for Secondary Schools: Protocol for the Education Against Tobacco Cluster Randomized Trial
Source: JMIR Res Protoc. 2019 Apr 11;8(4):e13508. doi: 10.2196/13508 (PMC6482400; doi:10.2196/13508)
Supplement: Multimedia Appendix 3 [file resprot_v8i4e13508_app3.docx]

**Statistical Analyses**

**Preparation curriculum for medical student volunteers:** Completeness and Feedback

We received mentor questionnaires from 11 of 13 medical schools (all except Heidelberg and Düsseldorf) and educator feedback (volunteering medical students) from 10 of 13 medical schools (all except Giessen, Heidelberg and Cologne). 100% of volunteering medical students for the study received training.

**Participant characteristics:**

| **Variable** | **Mentors (receive education for classroom visit)** | **Educators (deliver education to mentors)** |
| --- | --- | --- |
| Total | 324 | 63 |
| Number of mentors/educators per medical school | 29.5 (±16.7) | 6.3 (±3.4) |
| Age (Average/Median/Range) | 21.8 (±2.8) / 21 / 18 - 32 / 320 valid cases | 22.7 (±1.5) / 23 / 20 - 28 / 63 valid cases |
| Female | 217/322 (67.4%) | 37/61 (60.7%) |
| Male | 101/322 (31.4%) | 24/61 (39.3%) |
| Pre-clinical phase of medical school | 180/322 (55.9%) | 11/63 (17.5%) |
| Clinical phase of medical school | 142/322 (44.1%) | 52/63 (82.5%) |
| Non-smokers | 294/322 (91.3%) | 56/57 (98.2%) |
| Ex-smokers | 24/322 (7.5%) | - |
| Smokers | 4/322 (1.2%) | 1/57 (1.8%) |
| At least one parent not born in Germany: | 152/321 (47.4%) | 18/62 (29.0%) |

Percentage base: valid cases (number of questionnaires with valid answers)

**Participant perceptions:**

| **Variable** | **Mentors (receive education for classroom visit)** | **Educators (deliver education to mentors)** |
| --- | --- | --- |
| **What influence did the education/training have on yourself?** |  |  |
| Increased my motivation not to smoke | 1.5 (±0.8) / 320/ 92.2% | 1.5 (±1) / 63 / 85.7% |
| I learned new things about tobacco as a topic | 1.8 (±0.9) / 324/ 76.2% | 1.8 (±0.9) / 63 / 73.0% |
| It increased my awareness about the harms of tobacco | 1.8 (±0.8) / 323/ 84.8% | 1.8 (±1) / 61 / 85.2% |
| It increased my motivation to advice my future patients not to smoke | 1.3 (±0.6) / 323/ 94.4% | 1.2 (±0.4) / 61 / 100% |
| **How did you perceive the training?** |  |  |
| It was fun | 1.3 (±0.5) / 324/ 99.1% | 1.2 (±0.4) / 63 / 100% |
| It was interesting | 1.3 (±0.5) / 324/ 98.1% | 1.3 (±0.5) / 63 / 98.4% |
| I feel well prepared | 1.5 (±0.6) / 322/ 96.6% | 1.3 (±0.5) / 63 / 100% |
| I was able to train my didactic skills | 2 (±0.9) / 323/ 70.6% | 1.4 (±0.5) / 63 / 98.4% |
| **Global feedback** |  |  |
| Overall, the training made sense | 1.2 (±0.4) / 319/ 99.7% | 1.2 (±0.4) / 63 / 100% |
| I would recommend EAT to other medical students | 1.1 (±0.4) / 315/ 99.7% | 1.1 (±0.3) / 63 / 98.4% |

Used scale: 1=fully correct, 2=rather correct, 3=rather not correct, 4=not correct at all

Legend: mean (± standard deviation) / valid cases (percentage base) / percent of top two (1 or 2) related to valid cases

**Number of mentors vs. number of schools:**

| **Medical School** | **Number of educated mentors** | **Number of visited schools** |
| --- | --- | --- |
| Bochum | 38/324 (11.7%) | 5/72 (6,9%) |
| Bonn | 36/324 (11.1%) | 6/72 (8,3%) |
| Düsseldorf* | - | 11/72 (15,3%) |
| Erlangen | 20/324 (6.2%) | 11/72 (15,3%) |
| Essen | 20/324 (6.2%) | 5/72 (6,9%) |
| Freiburg | 40/324 (12.3%) | 4/72 (5,6%) |
| Hannover | 12/324 (3.7%) | 5/72 (6,9%) |
| Köln | 3/324 (0.9%) | 4/72 (5,6%) |
| Gießen | 50/324 (15.4%) | 5/72 (6,9%) |
| Göttingen | 11/324 (3.4%) | 1/72 (1,4%) |
| Regensburg | 48/324 (14.8%) | 4/72 (5,6%) |
| Tübingen | 46/324 (14.2%) | 5/72 (6,9%) |
| Heidelberg* | - | 6/72 (8,3%) |

*No questionnaires from mentors were handed in.

Comment: 72 schools (the complete intervention group) were visited by the mentors. But only 59 schools provided process evaluation feedback.

**Overall intervention classes with feedback by pupils: 208/260 (80.0%) and by teachers: 136/260 (52.3%)**

Intervention-questionnaire pupil’s characteristics:

Age: mean=12.8 / median=13 /range: 9 -17 / 4757 valid cases

Gender: male: 2109/4896 (43.1%); female 2213/4896 (45.2%); no answer: 574/4896 (11.7%); valid cases: 4322 (88.3%)

| **Variable** | **All pupils^1^** | **Male pupils** | **Female pupils** | **Not grammar school** | **Grammar school** | **Teachers^2^** | **Medical students (mentors)^3^** | **Med. students teaching mentors^4^** |
| --- | --- | --- | --- | --- | --- | --- | --- | --- |
| Overall number | 4896 | 2109/4322 (48.8%) | 2213/4322 (51.2%) | 2270/4896 (46.4%) | 2626/4896 (53.6%) | 141 | 324 | 63 |
| **Complete delivery of curriculum (yes)** | | | | | | | | |
| Presentation in great hall | 4822/4856 (99.3%) | - | - | - | - | - | - | - |
| All stations | 3988/4526 (88.1%) | 1708/1947 (87.7%) | 1842/2080 (88.6%) | 1866/2067 (90.3%) | 2122/2459 (86.3%) | 116/126 (92.1%) | 194/316 (61.4%) | 54/63 (85.7%) |
| Station 1 | 4699/4804 (97.8%) | - | - | - | - | - | - | - |
| Station 2 | 4628/4803 (96.4%) | - | - | - | - | - | - | - |
| Station 3 | 4788/4821 (99.3%) | - | - | - | - | - | - | - |
| Station 4 | 4548/4696 (96.8%) | - | - | - | - | - | - | - |
| Hung posters into the classroom* | 2646/4403 (60.1%) | - | - | - | - | 83/120 (69.2%) | - | - |

* From 208 school classes in 123 (59.1%) there was reported by the majority that the posters have been hung.

| **Curriculum Feedback** | **All pupils^1^** | **Male pupils** | **Female pupils** | **Not grammar school** | **Grammar school** | **Teachers^2^** | **Medical students (mentors)^3^** | **Med. students teaching mentors^4^** |
| --- | --- | --- | --- | --- | --- | --- | --- | --- |
| It was^1,2^/will be^3,4^ fun for the pupils | 1.7 (±0.8) / 4832 / 85.9% | 1.7 (±0.8) / 2087 / 85.9% | 1.7 (±0.8) / 2191 / 87.5% | 1.7 (±0.9) / 2229 / 84.2% | 1.7 (±0.8) / 2603 / 87.4% | 1.3 (±0.5) / 137 / 100% | 1.3 (±0.5) / 320 / 99.1% | 1.2 (±0.4) / 63 / 100% |
| It was^1,2^/will be^3,4^ interesting for the pupils | 1.5 (±0.8) / 4837 / 88.2% | 1.6 (±0.8) / 2088 / 87% | 1.5 (±0.7) / 2197 / 90.7% | 1.6 (±0.8) / 2236 / 86.2% | 1.5 (±0.8) / 2601 / 90% | 1.3 (±0.5) / 136 / 98.5% | 1.3 (±0.5) / 323 / 99.7% | 1.4 (±0.5) / 63 / 98.4% |
| It was^1,2^/will be^3,4^ easy to understand for the pupils | 1.4 (±0.6) / 4814 / 96.1% | 1.4 (±0.6) / 2077 / 95.2% | 1.3 (±0.6) / 2189 / 97.2% | 1.4 (±0.6) / 2226 / 94.8% | 1.3 (±0.6) / 2588 / 97.2% | 1.4 (±0.6) / 134 / 97% | 1.4 (±0.5) / 324 / 98.5% | 1.6 (±0.5) / 63 / 98.4% |
| They learned^1,2^ / will learn^3,4^ benefits of non-smoking that are new to them | 1.8 (±1) / 4819 / 76.1% | 1.9 (±1.1) / 2084 / 73.8% | 1.8 (±1) / 2189 / 79.5% | 1.8 (±1) / 2223 / 76.6% | 1.8 (±1) / 2596 / 75.7% | 1.4 (±0.6) / 133 / 93.2% | 1.4 (±0.6) / 314 / 94.3% | 1.2 (±0.6) / 63 / 96.8% |
| It motivated them^1,2^/will motivate^3,4^ them to be a non-smoker | 1.4 (±0.8) / 4814 / 90.6% | 1.5 (±0.8) / 2076 / 89% | 1.3 (±0.7) / 2185 / 92.8% | 1.4 (±0.8) / 2220 / 89.2% | 1.4 (±0.7) / 2594 / 91.8% | 1.6 (±0.7) / 131 / 93.9% | 1.5 (±0.5) / 316 / 98.4% | 1.4 (±0.6) / 63 / 95.2% |
| Overall, the program is age appropriate | 1.5 (±0.7) / 4809 / 90.4% | 1.5 (±0.7) / 2077 / 90% | 1.5 (±0.7) / 2183 / 91.8% | 1.5 (±0.7) / 2227 / 90.4% | 1.5 (±0.7) / 2582 / 90.3% | 1.3 (±0.5) / 135 / 98.5% | - | - |
| The pupils were able to engage a lot (OR medi-cal students engaged in training) | 1.9 (±0.8) / 4758 / 80.5% | 1.9 (±0.9) / 2055 / 80.1% | 1.9 (±0.8) / 2164 / 81.7% | 1.9 (±0.9) / 2182 / 80.7% | 1.9 (±0.8) / 2576 / 80.4% | 1.4 (±0.5) / 132 / 99.2% | - | - |

Used scale: 1=fully correct, 2=rather correct, 3=rather not correct, 4=not correct at all

Legend: mean (± standard deviation) / valid cases (percentage base) / percent of top two (1 or 2) related to valid cases

| **Feedback on medical students: They were…** | **All pupils^1^** | **Male pupils** | **Female pupils** | **Not grammar school** | **Grammar school** | **Teachers^2^** | **Medical students (mentors)^3^** | **Med. students teaching mentors^4^** |
| --- | --- | --- | --- | --- | --- | --- | --- | --- |
| Well prepared | 1.2 (±0.5) / 4854 / 97.5% | 1.3 (±0.5) / 2093 / 97.1% | 1.2 (±0.5) / 2206 / 98.5% | 1.2 (±0.5) / 2252 / 97.2% | 1.2 (±0.5) / 2602 / 97.8% | 1.1 (±0.3) / 138 / 99.3% | 1.7 (±0.7) / 321 / 89.4% | 1.6 (±0.7) / 63 / 87.3% |
| motivated | 1.3 (±0.6) / 4827 / 95.5% | 1.3 (±0.6) / 2082 / 95.3% | 1.3 (±0.6) / 2193 / 96.2% | 1.4 (±0.6) / 2229 / 94.8% | 1.3 (±0.6) / 2598 / 96.2% | 1.1 (±0.3) / 138 / 100% | 1.3 (±0.5) / 322 / 97.5% | 1.2 (±0.4) / 63 / 100% |
| On time | 1.3 (±0.6) / 4818 / 94.5% | 1.3 (±0.6) / 2074 / 94% | 1.3 (±0.6) / 2191 / 95.6% | 1.3 (±0.7) / 2223 / 93.6% | 1.3 (±0.6) / 2595 / 95.3% | 1.1 (±0.4) / 137 / 98.5% | 1.4 (±0.7) / 322 / 89.8% | 1.3 (±0.5) / 63 / 98.4% |
| Engaged with the students^1,2^ (with the teaching students^3,4^) | 1.3 (±0.6) / 4819 / 95.6% | 1.3 (±0.6) / 2080 / 95.6% | 1.3 (±0.6) / 2191 / 96.5% | 1.3 (±0.6) / 2226 / 95.8% | 1.3 (±0.6) / 2593 / 95.4% | 1.2 (±0.4) / 138 / 99.3% | 1.4 (±0.5) / 309 / 98.4% | 1.4 (±0.5) / 63 / 98.4% |
| Overall, they left a very good impression: | 1.3 (±0.6) / 4840 / 95.9% | 1.3 (±0.6) / 2083 / 96% | 1.3 (±0.5) / 2198 / 96.6% | 1.3 (±0.6) / 2234 / 95.4% | 1.3 (±0.6) / 2606 / 96.3% | 1.2 (±0.5) / 138 / 97.8% | 1.7 (±0.6) / 305 / 95.1% | 1.3 (±0.5) / 63 / 100% |

Used scale: 1=fully correct, 2=rather correct, 3=rather not correct, 4=not correct at all

Legend: mean (± standard deviation) / valid cases (percentage base) / percent of top two (1 or 2) related to valid cases

| **Feedback on Smokerface App specifically** | **All pupils^1^** | **Male pupils** | **Female pupils** | **Not grammar school** | **Grammar school** | **Teachers^2^** | **Medical students (mentors)^3^** | **Med. students teaching mentors^4^** |
| --- | --- | --- | --- | --- | --- | --- | --- | --- |
| Age-appropriate | 2.0 (±0.9) / 4803 / 71.9% | 2.0 (±1) / 2081 / 71.7% | 2.0 (±0.9) / 2174 / 72.5% | 2.0 (±1) / 2211 / 74.5% | 2.1 (±0.9) / 2592 / 69.7% | 1.5 (±0.7) / 132 / 92.4% | 1.3 (±0.5) / 322 / 97.5% | 1.3 (±0.6) / 63 / 93.7% |
| realistic | 2.6 (±1) / 4807 / 47.3% | 2.6 (±1) / 2081 / 45.9% | 2.5 (±0.9) / 2182 / 49% | 2.5 (±1) / 2217 / 50.7% | 2.6 (±0.9) / 2590 / 44.5% | 1.9 (±0.8) / 128 / 82% | 1.8 (±0.7) / 320 / 85% | 2.1 (±0.6) / 63 / 76.2% |
| Motivates the pupils not to smoke | 1.8 (±1) / 4820 / 77% | 1.8 (±1) / 2088 / 76.5% | 1.8 (±1) / 2181 / 78.5% | 1.8 (±1) / 2225 / 76.8% | 1.8 (±1) / 2595 / 77.1% | 1.8 (±0.8) / 128 / 83.6% | 1.7 (±0.7) / 320 / 90.3% | 1.7 (±0.6) / 63 / 90.5% |

Used scale: 1=fully correct, 2=rather correct, 3=rather not correct, 4=not correct at all

Legend: mean (± standard deviation) / valid cases (percentage base) / percent of top two (1 or 2) related to valid cases

**JUST PUPILS**

| **How did you like the presentation in the great hall?** | **All pupils** | **Male pupils** | **Female pupils** | **Not grammar school** | **Grammar school** |
| --- | --- | --- | --- | --- | --- |
| It was fun | 2 (±0.8) / 4820 / 78% | 1.9 (±0.8) / 2079 / 79.8% | 2 (±0.8) / 2192 / 77% | 1.9 (±0.9) / 2228 / 77.6% | 2 (±0.8) / 2592 / 78.4% |
| It was interesting | 1.6 (±0.8) / 4813 / 87.2% | 1.6 (±0.8) / 2075 / 86.6% | 1.6 (±0.8) / 2194 / 89% | 1.7 (±0.8) / 2225 / 85.2% | 1.6 (±0.7) / 2588 / 88.8% |
| It was easy to understand | 1.4 (±0.6) / 4798 / 96.2% | 1.4 (±0.6) / 2062 / 96.2% | 1.3 (±0.6) / 2188 / 96.7% | 1.4 (±0.6) / 2219 / 95.1% | 1.3 (±0.6) / 2579 / 97.1% |
| I learned benefits of non-smoking that were new to me | 1.9 (±1) / 4800 / 75.5% | 1.9 (±1.1) / 2069 / 72% | 1.8 (±1) / 2187 / 79.3% | 1.8 (±1) / 2211 / 76.8% | 1.9 (±1) / 2589 / 74.4% |
| It motivated me not to smoke. | 1.5 (±0.9) / 4797 / 87.5% | 1.6 (±0.9) / 2069 / 86.5% | 1.5 (±0.8) / 2179 / 89.7% | 1.5 (±0.9) / 2211 / 85.9% | 1.5 (±0.8) / 2586 / 88.8% |

Used scale: 1=fully correct, 2=rather correct, 3=rather not correct, 4=not correct at all

Legend: mean (± standard deviation) / valid cases (percentage base) / percent of top two (1 or 2) related to valid cases

| **What was discussed in it? (yes)** | **All pupils** | **Male pupils** | **Female pupils** | **Not grammar school** | **Grammar school** |
| --- | --- | --- | --- | --- | --- |
| Tobacco advertising | 4528/4630 (97.8%) | 1946/1992 (97.7%) | 2081/2123 (98%) | 2050/2111 (97.1%) | 2478/2519 (98.4%) |
| Influence on physical performance | 4530/4608 (98.3%) | 1962/1995 (98.3%) | 2075/2102 (98.7%) | 2039/2096 (97.3%) | 2491/2512 (99.2%) |
| Tablets were used | 4452/4682 (95.1%) | 1918/2016 (95.1%) | 2042/2139 (95.5%) | 2063/2163 (95.4%) | 2389/2519 (94.8%) |
| I made a selfie with a tablet | 2338/4765 (49.1%) | 1155/2048 (56.4%) | 904/2178 (41.5%) | 1146/2203 (52%) | 1192/2562 (46.5%) |
| My face was displayed via a beamer | 314/4724 (6.6%) | 177/2027 (8.7%) | 89/2164 (4.1%) | 187/2174 (8.6%) | 127/2550 (5%) |

Legend: number ‘yes’ / valid answers (percentage base) / (percent of ‘yes’ related to valid answers)

| **Feedback on Station 1** (Experiment with water bottle and a cigarette) | **All pupils** | **Male pupils** | **Female pupils** | **Not grammar school** | **Grammar school** |
| --- | --- | --- | --- | --- | --- |
| It was fun | 1.6 (±0.8) / 4813 / 88.4% | 1.6 (±0.8) / 2076 / 88.5% | 1.6 (±0.8) / 2189 / 89.1% | 1.6 (±0.8) / 2221 / 87.9% | 1.6 (±0.8) / 2592 / 88.8% |
| It was interesting | 1.5 (±0.7) / 4812 / 91% | 1.5 (±0.7) / 2075 / 90.8% | 1.4 (±0.7) / 2191 / 92.7% | 1.5 (±0.8) / 2218 / 89.5% | 1.4 (±0.7) / 2594 / 92.4% |
| It was easy to understand | 1.3 (±0.6) / 4779 / 95.9% | 1.3 (±0.6) / 2061 / 95.5% | 1.3 (±0.5) / 2179 / 97.1% | 1.3 (±0.6) / 2201 / 95.5% | 1.3 (±0.6) / 2578 / 96.3% |
| I learned benefits of non-smoking that were new to me | 1.8 (±1) / 4784 / 77.5% | 1.9 (±1) / 2065 / 75.8% | 1.8 (±0.9) / 2177 / 79.8% | 1.8 (±1) / 2193 / 77.9% | 1.8 (±1) / 2591 / 77.2% |
| It motivated me not to smoke. | 1.5 (±0.8) / 4790 / 87.9% | 1.5 (±0.9) / 2068 / 86.9% | 1.4 (±0.8) / 2175 / 90.1% | 1.5 (±0.9) / 2201 / 86.3% | 1.5 (±0.8) / 2589 / 89.3% |
| *The experiment with the water bottle and the cigarette was conducted (yes) | 4699/4804 (97.8%) | 2029/2074 (97.8%) | 2158/2198 (98.2%) | 2129/2215 (96.1%) | 2570/2589 (99.3%) |
| ***Knowledge question*** | **All pupils** | **Male pupils** | **Female pupils** | **Not grammar school** | **Grammar school** |
| **Which product contains more harmful substances?  (e-cigarettes). | 508/3887 (13.1%) | 234/1738 (13.5%) | 225/1719 (13.1%) | 250/1822 (13.7%) | 258/2065 (12.5%) |

Used scale: 1=fully correct, 2=rather correct, 3=rather not correct, 4=not correct at all

Legend: mean (± standard deviation) / valid cases (percentage base) / percent of top two (1 or 2) related to valid cases

*Legend: number ‘yes’ / valid answers (percentage base) / (percent of ‘yes’ related to valid answers)

**Legend: number of e-cigarettes quoted / valid answers (percentage base) / (percent of e-cigarettes quoted related to valid answers)

| **Feedback on Station 2** (Effects of smoking on the face) | **All pupils** | **Male pupils** | **Female pupils** | **Not grammar school** | **Grammar school** |
| --- | --- | --- | --- | --- | --- |
| It was fun | 1.7 (±0.8) / 4793 / 83.8% | 1.7 (±0.8) / 2074 / 83.9% | 1.7 (±0.8) / 2180 / 84.7% | 1.7 (±0.9) / 2209 / 84.3% | 1.7 (±0.8) / 2584 / 83.4% |
| It was interesting | 1.6 (±0.8) / 4782 / 86.2% | 1.6 (±0.8) / 2070 / 85.3% | 1.6 (±0.8) / 2176 / 88.3% | 1.6 (±0.8) / 2205 / 85.9% | 1.6 (±0.8) / 2577 / 86.4% |
| It was easy to understand | 1.3 (±0.6) / 4769 / 95% | 1.4 (±0.7) / 2064 / 93.7% | 1.3 (±0.6) / 2169 / 97% | 1.4 (±0.7) / 2199 / 93.9% | 1.3 (±0.6) / 2570 / 95.9% |
| I learned benefits of non-smoking that were new to me | 1.9 (±1) / 4762 / 75.1% | 1.9 (±1.1) / 2062 / 73% | 1.8 (±1) / 2165 / 77.2% | 1.8 (±1) / 2190 / 77.2% | 1.9 (±1) / 2572 / 73.3% |
| It motivated me not to smoke. | 1.5 (±0.9) / 4753 / 86.9% | 1.6 (±0.9) / 2059 / 85.6% | 1.5 (±0.8) / 2160 / 89.4% | 1.6 (±0.9) / 2188 / 85.6% | 1.5 (±0.8) / 2565 / 88% |

Used scale: 1=fully correct, 2=rather correct, 3=rather not correct, 4=not correct at all

Legend: mean (± standard deviation) / valid cases (percentage base) / percent of top two (1 or 2) related to valid cases

| **Feedback on Station 3** (Appearance aspects of smoking) | **All pupils** | **Male pupils** | **Female pupils** | **Not grammar school** | **Grammar school** |
| --- | --- | --- | --- | --- | --- |
| It was fun | 1.7 (±0.9) / 4777 / 83.5% | 1.7 (±0.9) / 2074 / 83.2% | 1.7 (±0.8) / 2182 / 84.3% | 1.7 (±0.9) / 2193 / 83.3% | 1.8 (±0.8) / 2584 / 83.6% |
| It was interesting | 1.6 (±0.8) / 4770 / 86.8% | 1.6 (±0.9) / 2072 / 86.1% | 1.6 (±0.8) / 2184 / 88.1% | 1.6 (±0.9) / 2187 / 86.4% | 1.6 (±0.8) / 2583 / 87.1% |
| It was easy to understand | 1.4 (±0.7) / 4749 / 93.4% | 1.4 (±0.7) / 2063 / 92.6% | 1.4 (±0.6) / 2173 / 95.1% | 1.4 (±0.7) / 2176 / 92.6% | 1.4 (±0.7) / 2573 / 94.2% |
| I learned benefits of non-smoking that were new to me | 1.8 (±1) / 4739 / 77% | 1.9 (±1.1) / 2060 / 74.7% | 1.8 (±1) / 2169 / 79.4% | 1.8 (±1) / 2190 / 77.3% | 1.9 (±1) / 2572 / 76.1% |
| It motivated me not to smoke. | 1.6 (±0.9) / 4747 / 86.1% | 1.6 (±0.9) / 2063 / 85.2% | 1.5 (±0.8) / 2168 / 88.1% | 1.6 (±0.9) / 2171 / 84.5% | 1.5 (±0.9) / 2576 / 87.4% |

Used scale: 1=fully correct, 2=rather correct, 3=rather not correct, 4=not correct at all

Legend: mean (± standard deviation) / valid cases (percentage base) / percent of top two (1 or 2) related to valid cases

| *Which appearance aspects were discussed? (yes)* | **All pupils** | **Male pupils** | **Female pupils** | **Not grammar school** | **Grammar school** |
| --- | --- | --- | --- | --- | --- |
| Cellulitis | 1941/2834 (68.5%) | 785/1143 (68.7%) | 938/1372 (68.4%) | 942/1275 (73.9%) | 999/1559 (64.1%) |
| Saggy skin and breasts | 3481/4129 (84.3%) | 1507/1765 (85.4%) | 1602/1917 (83.6%) | 1570/1848 (85%) | 1911/2281 (83.8%) |
| Pimples | 4599/4676 (98.4%) | 1977/2016 (98.1%) | 2126/2147 (99%) | 2088/2125 (98.3%) | 2511/2551 (98.4%) |
| Stunted body growth | 3119/4012 (77.7%) | 1379/1733 (79.6%) | 1407/1851 (76%) | 1452/1768 (82.1%) | 1667/2244 (74.3%) |
| Stunted lung growth | 2512/3557 (70.6%) | 1142/1579 (72.3%) | 1090/1582 (68.9%) | 1267/1591 (79.6%) | 1245/1966 (63.3%) |
| Adiposity | 2551/3790 (67.3%) | 1088/1636 (66.5%) | 1207/1743 (69.2%) | 1113/1623 (68.6%) | 1438/2167 (66.4%) |
| Impotence | 2586/3390 (76.3%) | 1281/1583 (80.9%) | 1035/1445 (71.6%) | 1141/1454 (78.5%) | 1445/1936 (74.6%) |
| *Which of those is most important to you?* | Stunted lung growth 962/4267 (22.5%)  Pimples 842/4267 (19.7%)  Adiposity 766/4267 (18%)  Saggy skin and breasts 554/4267 (13%)  Stunted body growth 522/4267 (12.2%)  Impotence 502/4267 (11.8%)  Cellulitis 119/4267 (2.8%) | most frequent denomination:  Pimples:  402/1816 (22.1%) | most frequent denomination:  Stunted lung growth: 559/1999 (28%) | most frequent denomination:  Pimples:  440/1891 (23.3%) | most frequent denomination:  Stunted lung growth:  583/2376 (24.5%) |
| *Effect on blood pressure* **(it rises)** | 2260/3064 (73.8%) | 955/1355 (70.5%) | 1077/1390 (77.5%) | 886/1324 (66.9%) | 1374/1740 (79%) |
| Was water pumped through a pipe via a syringe? *(Yes)* | 3822/4497 (85%) | 1653/1944 (85%) | 1753/2073 (84.6%) | 1919/2080 (92.3%) | 1903/2417 (78.7%) |

Legend: number ‘yes’ or quoted / valid answers (percentage base) / (percent of ‘yes’ or quoted related to valid answers)

| **Feedback on Station 4** (Own experiences with smoking and how to reject a cigarette) | **All pupils** | **Male pupils** | **Female pupils** | **Not grammar school** | **Grammar school** |
| --- | --- | --- | --- | --- | --- |
| It was fun | 1.7 (±0.9) / 4730 / 85.7% | 1.6 (±0.9) / 2052 / 85.4% | 1.6 (±0.8) / 2161 / 87% | 1.6 (±0.9) / 2191 / 85.7% | 1.7 (±0.8) / 2539 / 85.7% |
| It was interesting | 1.6 (±0.8) / 4721 / 87% | 1.6 (±0.9) / 2050 / 86% | 1.5 (±0.8) / 2164 / 88.9% | 1.6 (±0.9) / 2187 / 86.5% | 1.6 (±0.8) / 2534 / 87.4% |
| It was easy to understand | 1.4 (±0.7) / 4706 / 94.3% | 1.4 (±0.7) / 2042 / 93.2% | 1.3 (±0.6) / 2157 / 96.3% | 1.4 (±0.7) / 2183 / 93.8% | 1.3 (±0.6) / 2523 / 94.8% |
| I learned benefits of non-smoking that were new to me | 1.9 (±1) / 4691 / 75.5% | 1.9 (±1.1) / 2042 / 73.1% | 1.8 (±1) / 2144 / 78.2% | 1.8 (±1) / 2167 / 77.3% | 1.9 (±1) / 2524 / 73.9% |
| It motivated me not to smoke. | 1.6 (±0.9) / 4689 / 85.1% | 1.6 (±0.9) / 2037 / 83.9% | 1.5 (±0.9) / 2144 / 87.4% | 1.6 (±0.9) / 2166 / 83.9% | 1.5 (±0.9) / 2523 / 86.1% |

Used scale: 1=fully correct, 2=rather correct, 3=rather not correct, 4=not correct at all

Legend: mean (± standard deviation) / valid cases (percentage base) / percent of top two (1 or 2) related to valid cases

| *What was done/discussed?* | **All pupils** | **Male pupils** | **Female pupils** | **Not grammar school** | **Grammar school** |
| --- | --- | --- | --- | --- | --- |
| I was asked for my own experiences with smoking *(Yes)*. | 3267/4280 (76.3%) | 1407/1872 (75.2%) | 1535/1958 (78.4%) | 1342/1896 (70.8%) | 1925/2384 (80.7%) |
| We talked about how to reject a cigarette *(Yes).* | 1782/3990 (44.7%) | 805/1752 (45.9%) | 790/1810 (43.6%) | 892/1766 (50.5%) | 890/2224 (40%) |
| We used cards to learn the four phases of addiction *(Yes).* | 4312/4495 (95.9%) | 1843/1938 (95.1%) | 2027/2079 (97.5%) | 1910/2016 (94.7%) | 2402/2479 (96.9%) |
| *I know the four phases of addiction (Yes).* | 3828/4260 (89.9%) | 1633/1841 (88.7%) | 1820/1976 (92.1%) | 1600/1858 (86.1%) | 2228/2402 (92.8%) |

Legend: number ‘yes’ / valid answers (percentage base) / (percent of ‘yes’ related to valid answers)

**JUST TEACHERS**

COMPLETED QUESTIONNAIRES: 67 / 141 (47.5%) Questionnaires are completely answered (without ‘no answer’ at all).

At only 20 of the 141 questionnaires (14.2%) there are 3 or more questions missing.

| **Global feedback** |  |
| --- | --- |
| *Overall, the intervention made a lot of sense. | 1.2 (±0.5) / 137 / 98.5% |
| *I would recommend the program to other schools. | 1.2 (±0.5) / 137 / 97.1% |
| *The presentation in the great hall was very good. | 1.7 (±0.8) / 103 / 89.3% |
| *The classroom intervention was very good. | 1.3 (±0.5) / 130 / 98.5% |
| The aula presentation took X minutes | mean=46.3 min / median=40 min /range: 30 – 90 min  88/141 (62.4%) valid answers |
| The classroom intervention took X minutes | mean=90.1 min / median=90 min /range: 50 – 120 min  119/141 (84.4%) valid answers |

*Legend: mean (± standard deviation) / valid cases (percentage base) / percent of top two (1 or 2) related to valid cases
